# The Tubercular Negro—An Unsolved Problem

**Published:** 1912-06

**Authors:** W. C. Bryant

**Affiliations:** Clarksville, Ga.


### THE TUBERCULAR NEGRO—AN UNSOLVED PROBLEM.

By W. C. Bryant, M. D., Clarksville, Ga.

Paper read at the meeting of the Ninth District Medical Society at Gainesville, Ga., March, 1912, by Dr. W. C. Bryant, of Camp Yonah Sanatorium.

Paper discussed by majority of members, and upon motion of Dr. L. G. Hardman, of Commerce, a committee was appointed to draft formal resolutions approving the position taken by Dr. Bryant.

It is very much easier to state a subject for discussion than to dig up and collate the pertinent facts bearing on the subject itself.

This stubborn truism stared me boldly and disconcertingly in the face when I realized that in choosing a theme for my paper I had selected one, that in the very beginning, carried me beyond the bounds usually recognized as the proper limit for a medical man to venture.

But, as Fate—or Circumstance—has fixed it, I am a sort of homogenous mixture of physician and farmer, which fact, I take it, entitles me to wander farther afield than would be ordinarily allowed and I will ask that you pardon me if I ignore the usual lines of demarkation and will follow me with my subject although it carry us out and into the field of economics, anthropology and even into the bog of sociology.

My subject is one that is of vital interest to all, and especially to us of the South, but in the fifteen minutes allowed me I can only hope to say enough to awaken you and put you to thinking upon it more than you have thought heretofore.

The comparatively recent emergence of the negro from his uncivilized state and habitat into our civilization has added emphasis to the fact that tuberculosis is a product of civilization, for it is a well known fact that, up to the time that freedom and that abominable outrage that was committed against our people at the same time, the right of franchise, tuberculosis was practically unknown among the Southern slaves.

With his freedom came liquor, licentiousness, dirt and disease and—this is the problem we are "up against" now.

The diseased negro is a greater menace to society, if possible, than the morally vicious negro, and the sooner steps are taken, the better for our people.

Eliminate the problem of every vestige of sympathy and humanitarianism and the primal law of self-protection will force us, sooner or later, to enact laws that will effectually protect us from the unsanitary "Brother in Black."

The lure of the city has caused the negro to turn his back on rural districts and his presence in these congested centers of population is a growing menace to public health and goes to make an economic problem the solution of which will call for many a hard-earned dollar to be taken from the pockets of already over-burdened tax-payers.

The conscientious physician may advise and warn and do, as he always does, more for his community than any ten men, or any number of Foreign Missionary Societies in it, but, unless the strong arm of the law is invoked and speedily interposed, the problem grows more difficult of management each day, and the pity of it is that innocent lives, thousands of them, will be the penalty of delay.

We are told that our main remedy is education and evolution. This I grant, but I want to say that an officer armed with a warrant is an educator who can get results quicker than any other agency and can cause a shiftless, unsanitary negro to evolute amazingly.

I will not take your time in giving statistics as to the relative frequency of tuberculosis appearing in the white and black races, nor of the many points of contact which amounts so often to infection. The problem is well known by you, but the—"What are you going to do about it," is calling in a thousand ways for an answer.

The medical profession has been and is still doing what they can, but this is a problem the solution of which rests entirely with society—using the word in its broadest sense, and it is our duty, as physicians, to arouse public sentiment in this matter.

The crying need of our State is a compulsory registration of every case of tuberculosis, and every other communicable disease as to that matter, but this is only the first step in the work before us.

We need a central, or State Board of Health, in which those who work should receive sufficient compensation and then be required to devote all their time to the work.

The citizens of our state are now being taxed to support a State Board of Health that is falling far short of what it was intended to be for, it is a deplorable but well known fact that it is the storm center of political dissention and factional strife.

The little sanatorium for the tubercular at Alto may be doing all that it can, under the circumstances, but, unless the law creating it is amended and its scope broadened and made more comprehensive, it can never amount to a real factor in our vital statistics.

We understand that no provision is made for the admission of negroes there—a glaring evidence of short-sightedness—aside from the moral and legal injustice done.

What we need, in my humble opinion, is a County Board of Health in every county in the State, clothed with ample authority and charged with the duty of looking after and providing for the care of those within the county who may be stricken with a contagious or infectious disease and who are unable to care for themselves.

This Board should act in conjunction, in fact, be a part of the State Board of Health. Its officers should be composed of physicians legally practicing within the county, and of recognized ability, to be appointed by the Governor upon recommendation of the Grand Jury.

This Board should be empowered to elect a physician who should receive a reasonable compensation for his entire time. What is now the "County Poor Farm" should be made into a small but modern sanatorium and provided with a competent trained nurse.

The details of this could be easily worked out, and I believe that even the blindest and stingiest man in your county could be shown that, from an economic standpoint, some sort of a plan working along the lines suggested should be attempted. I hope that when you come to discuss what, on account of the short time allowed me, I have only been able to hint at, you will speak your minds freely, for I am sure that all of you will agree with me that the time has come when something more in keeping with modern science and the needs of society must take the place of our antiquated methods of dealing with public health.

With the knowledge that is now in our possession, it is a crime against society that so many die each day from diseases that are known as "preventable," and as increased knowledge carries with it a proportionate increase of responsibility, I fail to see how we can retain our self-respect if we continue to dodge our responsibility in this matter.

If some such arrangement as I have roughly outlined should be adopted great good would result to all our people—rich and poor—as the evoluted "poor farm" would become the center from which advanced medical knowledge would rediate. The officer in charge would work in conjunction with the general practitioner, aiding him by doing the laboratory work which the hard worked practitioner rarely has the time or equipment to do.

That there will be opposition to the plan suggested goes without saying, but this will not deter those who really have the good of humanity at heart, and I predict that something along the line I suggest will be worked out in a few years.

We hear a great deal about the "conservation" of one thing and another lately, but I very much fear that the main object of conservation, the conservation of our natural and other resources to the end that those who are to follow us may be conserved, is being overlooked by many of our so-called conservationists.

Again, gentlemen, let me ask that you "think on these things."

